# The Effect of Dataset Imbalance on the Performance of SCADA Intrusion Detection Systems

**DOI:** 10.3390/s23020758

**Published:** 2023-01-09

**Authors:** Asaad Balla, Mohamed Hadi Habaebi, Elfatih A. A. Elsheikh, Md. Rafiqul Islam, F. M. Suliman

**Affiliations:** 1 Department of Electrical and Computer Engineering, International Islamic University Malaysia, Kuala Lumpur 53100, Malaysia; 2 Department of Electrical Engineering, College of Engineering, King Khalid University, Abha 61421, Saudi Arabia

**Keywords:** IDS, ICS, SCADA, imbalanced datasets, cyber security

## Abstract

Integrating IoT devices in SCADA systems has provided efficient and improved data collection and transmission technologies. This enhancement comes with significant security challenges, exposing traditionally isolated systems to the public internet. Effective and highly reliable security devices, such as intrusion detection system (IDSs) and intrusion prevention systems (IPS), are critical. Countless studies used deep learning algorithms to design an efficient IDS; however, the fundamental issue of imbalanced datasets was not fully addressed. In our research, we examined the impact of data imbalance on developing an effective SCADA-based IDS. To investigate the impact of various data balancing techniques, we chose two unbalanced datasets, the Morris power dataset, and CICIDS2017 dataset, including random sampling, one-sided selection (OSS), near-miss, SMOTE, and ADASYN. For binary classification, convolutional neural networks were coupled with long short-term memory (CNN-LSTM). The system’s effectiveness was determined by the confusion matrix, which includes evaluation metrics, such as accuracy, precision, detection rate, and F1-score. Four experiments on the two datasets demonstrate the impact of the data imbalance. This research aims to help security researchers in understanding imbalanced datasets and their impact on DL SCADA-IDS.

## 1. Introduction

Supervisory control and data acquisition (SCADA) systems are used in a variety of industries to monitor and control industrial processes, such as manufacturing, energy, and transportation. They consist of a central computer or server that communicates with a network of devices, such as sensors and control systems, to collect data and control various processes [1]. For example, in the energy industry, a SCADA system might be used to monitor and control the flow of electricity through a power grid. Sensors within the grid would collect data on the flow of electricity, and the SCADA system would use that data to adjust the grid in real-time to ensure that the electricity is distributed efficiently and safely.

In recent years, there has been an increase in the integration of IoT in SCADA systems. An IoT platform connects billions of devices, including PLCs, actuators, and intelligent electronic devices (IEDs) of industrial control systems (ICS) [1]. SCADA systems benefit greatly from IoT in automation, improved monitoring, and data collection. The Internet of Things platform has enabled the modern industry to efficiently monitor and control physical systems (different hardware and machinery), resulting in intelligent data collecting, processing, and highly effective and successful business management. The use of IoT in SCADA is impossible unless the isolation of SCADA networks is broken, as these devices interact via the public internet. This advancement enhances the performance of SCADA networks and introduces new vulnerabilities and hazards for cyberattacks.

Security researchers in industry and academia have created many types of intrusion detection systems (IDSs) to improve the security of SCADA networks. Some of them are classic rule-based intrusion detection systems (IDS) while others have created anomaly detection solutions using machine learning (ML) and deep learning (DL) algorithms. A good SCADA dataset for training and evaluating ML/DL models is required to construct an efficient and intelligent IDS.

When we examined the SCADA intrusion detection dataset attentively, we noticed data imbalances caused by many regular traffic samples compared to only a few anomalies. This is because normal operations produce more data compared to cyberattacks. This is highlighted also in [2], in which the authors analyzed the CICIDS2017 dataset. When utilizing machine learning to detect attacks as anomalies, we must address data imbalance to develop a classifier that accurately distinguishes between regular and abnormal traffic. A classifier will have a difficult time detecting anomalies without overfitting when these dataset imbalances are not considered [3]. Historically, there have been few reliable and publicly available datasets. Those that are now available have been chastised for being out of date, lacking sufficient labeling, and including flaws that do not exist in real-world applications [4].

This is due to the following factors: (a) SCADA is concerned with critical infrastructure and industrial control systems. As a result, information regarding how these systems work would be inaccessible. (b) Due to privacy concerns, companies and governments do not share SCADA datasets. These systems hold sensitive and secret data. (c) Such data could be a gold mine for hostile individuals, companies, or state actors. We focus on this problem in this study and demonstrate the impact of data imbalances on intrusion detection using machine learning techniques. The motivation is that there is a critical need to design effective IDS models for the following reasons:

- Cyberattacks against SCADA networks and industrial control systems are on the rise;
- Increased risks and vulnerabilities are associated with IoT integration in SCADA systems (network leaks);
- To identify an effective route for building ML/DL IDS models in the absence of a reliable dataset.

For these reasons, we consider the Morris power and the CICIDS2017 datasets comprising power grid substation network traffic. We also examine CNN-LSTM algorithms and the handling of data imbalances for improving anomaly detection. We raise the following research question: Is there a difference in the performance of a machine learning model when the data is balanced versus unbalanced, and if so, what is the difference in metrics such as accuracy, precision, recall, and F1-score?

This paper is organized as follows: The next section discusses the relevant research based on the research questions. Then, in Section 3, we explain the methodology employed in this research, which includes a review of various techniques for dealing with imbalanced datasets. Section 4 contains the experiment results and answers to the research questions. We offer recommendations and suggestions for overcoming the difficulties highlighted in Section 5. Finally, we conclude the paper.

## 2. Related Works

In the existing literature, security researchers have widely studied the topic of dataset imbalance. This section discusses the related work that deals with imbalanced datasets in IDS models for SCADA systems. The publications discussed were chosen based on the following criteria: (a) they were published between 2018 and 2022, (b) they are review papers or research papers published only in journals, and (c) they contained the terms “IDS” and “imbalanced dataset.” These articles were gathered from ScienceDirect, Wiley Online Library, and Google Scholar. Before examining the related works, some concepts, such as SCADA systems, intrusion detection systems, data imbalance, undersampling, and oversampling, must be defined.

SCADA systems are control systems comprised of two major components: field devices, such as remote terminal units (RTUs) and programmable logic controllers (PLCs), and human machine interface (HMI). These systems monitor and control properties across a large geographic area as well as automate and control industrial operations. IoT devices collect data in modern SCADA systems, and data are transmitted via the public internet network. As a result, intrusion detection systems (IDSs) were created to secure such systems [5].

Intrusion detection systems (IDSs) are classified as either network-based or host-based. They operate in three modes: rule-based, anomaly detection, and hybrid. Machine learning (ML) and deep learning (DL) models are at the heart of these anomaly detection systems. A good dataset is required for training such models, which can be acquired from an entire SCADA system or a simulated system utilizing testbeds. One of the difficulties that security researchers face while constructing an ML/DL model is data imbalance.

When one class has a higher percentage than another, data imbalance occurs [6]. This is interpreted as a problem since it introduces bias into the results of ML/DL models. The model intends to classify an input as being in the majority. There have been published works on two primary solutions to this problem: undersampling and oversampling. Undersampling is a technique for lowering the proportion of the majority class. Oversampling, on the other hand, increases the minority class’s percentage by randomly reproducing it. The following section will go through works that employed these two approaches to resolve dataset imbalance.

The real network traffic data from the SCADA system contains a substantial amount of regular traffic and a minor amount of irregular traffic, which is a classic imbalanced data categorization challenge. Other datasets are created by simulations in which attack scenarios are manually injected into the system, resulting in more attacks than usual, such as in the case of the BoT–IoT dataset [7]. Although the prediction accuracy of some majority classes improves when the total error is minimized in this scenario, the prediction accuracy of minority classes is typically poorer.

Random undersampling (RUS) and random oversampling (ROS) are two popular sampling approaches. In network intrusion detection, the unbalanced ratio (IR) of various traffic data is extremely high. When the RUS method is employed, crucial information may be lost [8]. Using only the ROS strategy, on the other hand, will allow the classifier to learn a large amount of information, resulting in overfitting [9].

KDD99 employs a random forest (RF) with clustering undersampling to address the unbalanced dataset problem [10]. The model has two detection levels. The first level classifies the data instance as an assault or normal. The second level decides the attack based on the output of level one attacks. Because the attack types are uneven, the undersampling technique is applied at this stage. To undersample the majority class, [11] applied clustering with instance selection. The technique was then experimentally evaluated using affinity propagation and k-means algorithms, and three alternative instance selection algorithms (IB3, DROP3, and GA) were independently coupled for performance comparisons. The authors in [12] used clustering undersampling to create an IDS with NSL-KDD and UNSW-NB15. The k-means algorithm was used to construct new clusters for the majority class.

The authors in [13] employed an adaptive synthetic oversampling method (ADASYN) to address dataset imbalance. The detection approach in this study is a hybrid of a sparse autoencoder and random forest. To overcome the imbalance data problem, [14] coupled SMOTE and edited nearest neighbors (SMOTE-ENN) at the preprocessing stage. The data were then transformed into visuals for feature extraction with CNN.

In [15], the authors utilized a different technique to balance the data depending on the proportions of the classes in the sample. A CNN served as the foundation for the IDS model. To address the data imbalance problem, their research employed a combination of undersampling and oversampling strategies. They used the SVM and random forrest methods for the classification.

## 3. Methodology

This section provides the steps needed to answer the research questions and to achieve the goal of this paper, which is to understate the impact of datasets imbalance on the development of IDS in SCADA systems. This section is organized as follows: firstly, training the CNN-LSTM with imbalanced datasets; secondly, training the model with balanced training data; thirdly, a brief description of the datasets used in this paper; and finally, the experiment environment and settings are discussed.

### 3.1. CNN-LSTM with Imbalanced Datasets

**Figure 1:**
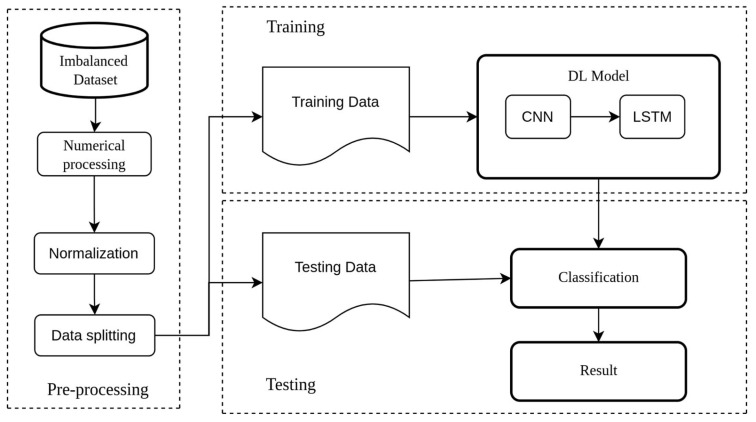
The flowchart of training the CNN-LSTM model with imbalanced data.

Figure 1 shows the flow of this experiment. The unbalanced SCADA datasets were adjusted using the MinMaxScaler with 70% of the data used for training and 30% for testing. This experiment consists of three steps, which are as follows:

1. Dataset preprocessing. In this step, categorical features are converted to numerical features. The data values are then normalized between 0 and 1;
2. Training and testing. The CNN-LSTM model is developed, and the best parameters for training the dataset are chosen;
3. Evaluation stage. The model’s performance is evaluated using metrics, such as accuracy, recall, and F1-score.

The Morris power dataset comprises only one categorical data, the “marker,” for the preprocessing stage (Normal, Attack). Using binary encoding, this attribute was converted into numerical data. Normal was “0,” while Attack was “1.” In the same way, the multiclass in the CICIDS2017 dataset was converted into Normal and Attack as well. Then, using the min–max function, all the values were normalized between 0 and 1, see Equation (1).

xi = (xi − Min)/(Max − Min)(1)

Any instance that contained missing values was removed as well as any feature with the same value for more than 80% of all records.

### 3.2. CNN-LSTM with Balanced Datasets

**Figure 2:**
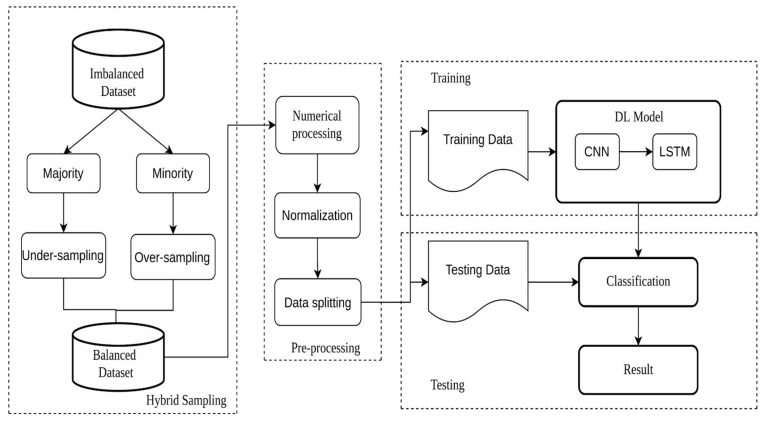
The flowchart of balancing the datasets and training the CNN-LSTM model with balanced data.

Figure 2 illustrates the flowchart of balancing the datasets and training the model. In Experiments 2, 3, and 4, the dataset was divided based on its majority and minority classes. To balance the dataset, the majority class was undersampled and the minority class was oversampled. In undersampling, there are three techniques:

- Methods that select records to keep, such as near miss under sampling and condensed nearest neighbor rule for undersampling;
- Methods that select instances to delete, including Tomek links for undersampling and edited nearest neighbors rule for undersampling;
- Combinations of both techniques. One-sided selection and neighborhood cleaning rule are examples of this approach;
- In oversampling, there are five techniques:
- Random oversampling;
- Synthetic minority oversampling technique (SMOTE);
- Borderline–SMOTE;
- Borderline oversampling with SVM;
- Adaptive synthetic sampling (ADASYN).

### 3.3. Datasets Description

**Figure 3:**
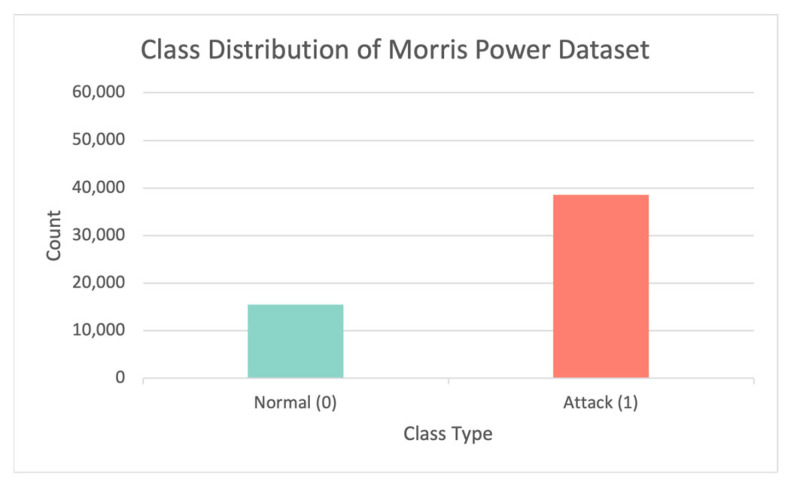
The percentage of attack records compared to normal instances in the Morris power dataset.

The Morris power dataset is imbalanced, as shown in Figure 3, which shows the class distribution in the dataset. To understand the dataset, principal component analysis (PCA) was used to visualize the data. From the visualization, we can determine whether a clear pattern can be seen and if so, determine which ML/DL model is most appropriate. The output of the PCA can provide a clear picture of the overall dataset. Although PCA reduces the dimensionality of the dataset, hence losing some data. It simply groups together features that are strongly correlated. We need an accuracy of approximately 70% to understand the overall dataset; we do not need to see every street to know the city.

**Figure 4:**
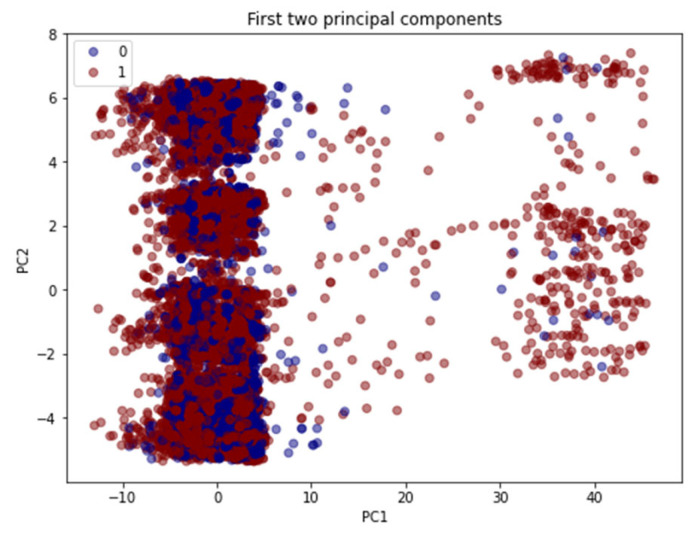
The result of conducting principal component analysis (PCA) on the Morris power Ddataset.

The original dataset contains 129 features. First, the data were cleaned by removing columns that had low variance. There were 36 columns with the same data across 80% of the rows, which would not contribute to the model performance. The SCADA dataset was also checked for any missing values. Using PCA, the remaining 93 features were reduced to 6 principal components. The first two principal components are visualized in Figure 4; it shows that there is no distinct boundary, and therefore it needs a more complicated model, such as a neural network, to define these boundaries. The first principal component represents around 27% of the data, and the 6th principal component represents only 3.47%. It is understood that these components represent around 71% of the data, but we do not need to see all 100% of the data to see a pattern.

**Figure 5:**
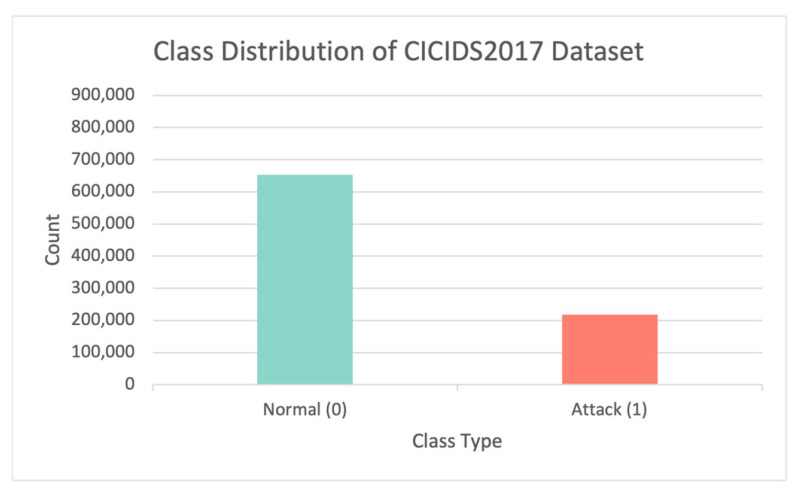
The class distribution in the CICIDS2017 dataset.

The Canadian Cyber Security Institute collected and assembled the CICIDS2017 dataset with the help of the B-Profile system at the end of 2017 [16]. The dataset contains 2,830,473 network traffic samples, with benign traffic accounting for 80.30 percent and attack traffic accounting for 19.70 percent. The categories include the most prevalent attacks, such as DoS, DDoS, Botnet, PortScan, web attacks, etc. The dataset collects 84 features from the generated network traffic, with the multiclass label being the last column. Furthermore, compared to publicly available datasets from 1998 to 2016, this dataset entirely fits the 11 performance evaluation criteria. The CICIDS2017 dataset is divided similarly to the UNSW-NB15 dataset. Figure 5 depicts the CICIDS2017 data distribution for each class.

### 3.4. Experiment Settings

Four experiments were conducted to determine the effect of dataset imbalance. In the first one, CNN-LSTM detected intrusions using imbalanced data. In the second experiment, the data were balanced using undersampling only. The model was trained with balanced data using an oversampling approach in the third experiment. A hybrid balancing technique was used in the fourth experiment, undersampling the majority class and oversampling the minority class. Next, the CNN-LSTM model was used to detect anomalies in the dataset.

Google Colab was used for these experiments because it is easy to use and provides GPU access to improve the model’s training. Average values were reported for each experiment after it was conducted several times. The deep learning model was built with the TensorFlow, Pandas, and Keras frameworks. The measures we used to assess the performance of these experiments are described in the next section.

The evaluation metrics used in these experiments are briefly discussed in this section. All experiments evaluate the model based on accuracy (ACC), recall, precision, and F1-score. For binary and multiclass classification problems, accuracy is the most common performance metric. An IDS accuracy rate measures how accurately it detects normal or abnormal network traffic [13]. The true positive rate (TPR) is the ratio of correctly predicted network anomalies and the total number of network anomalies. The TPR is called recall or sensitivity. The precision rate is an indicator of accuracy, which indicates the proportion of the number of positive cases correctly classified by the classifier to the number of positive cases. The F1-score is the weighted harmonic average of precision and recall, which is quite effective for the imbalanced classification problem.

## 4. Results and Analysis

In this section, we present the numerical results of our experiments in detecting the attacks through different dataset-balancing approaches, as mentioned in the previous subsection. The main goal of this section is to provide the answers to the research questions. We discussed the details of the study, including the evaluation metrics used and the outcome. We used the CNN-LSTM model to classify attacks in SCADA network data to answer the research questions.

### 4.1. Results of the Imbalanced Datasets

Initially, we compared dataset performance without employing any balancing strategy. The datasets were divided into two groups for binary classification: benign and attack; this is illustrated in Table 1. Table 2 presents the exact values for the evaluation metrics, which are the accuracy, precision, recall, and F1-score. In the Morris power dataset, the accuracy was the highest when the features containing the same value in more than 70% of the instances were removed. On the other hand, the accuracy of the CICIDS2017 dataset remained constant with different thresholds. However, accuracy is not the best measure to evaluate performance in intrusion detection scenarios with an imbalanced dataset. Because a large portion of training data is regular traffic, the algorithms are skewed toward estimating all data as usual and disregarding the small percentage of attack events [8].

The CICIDS2017 performance metrics are much better than those of the Morris power dataset, with approximately 97% and 75% for F1-scores. After comparing four values for the variance threshold, we continued our research using the threshold of 70%.

### 4.2. Results of the Balanced Datasets

#### 4.2.1. Undersampling

The second phase of our experiment was to sample the data using undersampling techniques, initially, balancing the CIC-IDS2017 dataset with random undersampling and CNN-LSTM. The selection of features was determined by the ANOVA F-value, which selected the highest score. The dataset was balanced using undersampling algorithms, such as the random undersampler (RUS), one-sided selection, and near miss algorithms. The data were then divided into training and testing segments with a 70:30 split. The CNN-LSTM model was trained with balanced datasets.

The results of balancing the Morris power dataset are shown in Table 3. The attack class was cut by 35.3 percent when a random undersampler was used. The one-sided selection approach reduced the majority class by only 2.3 percent. The near miss method produced the best results, reducing the attack class by half. Table 4 shows the binary classification result using the balanced Morris Power dataset. Although the random undersampler produced a greater F1-score than the other algorithms, the near miss approach produced a higher F1-score, which is the primary metric in our research. The performance improved by 9 percent compared to the unbalanced dataset. The unbalanced dataset has an F1-score of 57% while the balanced dataset has an F1-score of 66%.

Compared to the Morris power dataset, the CICIDS2017 dataset is 18 times larger in size, and the sampling process took a long time. Table 5 and Table 6 display the results of applying the undersampling method to balance this large dataset. As far as a balanced dataset was concerned, the near miss algorithm delivered the best results. To a maximum of 99.34 percent, the model’s performance was boosted by 2%. The random undersampler achieved 96 percent while one-sided selection generated an F1-Score of 97.67%. Overall, the performance of the CICIDS2017 datasets is excellent.

#### 4.2.2. Oversampling

This section describes the third experiment, which only used oversampling approaches to balance the datasets. Random oversampler (ROS), the synthetic minority oversampling technique (SMOTE), and adaptive synthetic sampling were the algorithms used to balance the data (ADASYN). The remaining steps were the same as detailed in Section 3.2.

Table 7 displays the outcomes for the Morris power dataset; employing ROS, the minority class doubled in size. SMOTE oversamples the normal class, resulting in a nearly balanced dataset. ADASYN performed well when oversampling the minority category but did not perform optimally. In terms of performance, the accuracy of all algorithms is around 71%. The difference is evident in the other metrics; for example, the SMOTE algorithm performed best in the F1-score, scoring 64%; the detailed findings are shown in Table 8.

In the CICIDS2017 dataset, the SMOTE algorithm outperformed ROS and ADASYN in oversampling the minority class; Table 9 provides the actual values. Table 10 displays the outcome of the CNN-LSTMM binary classification with the oversampled dataset. The drop in performance when the ADASYN sampling approach is applied is apparent in these results. The accuracy declined from 99.40% when SMOTE was used and to 93.62% when ADASYN was used, and the F1-score dropped from 99.46% to 93.25%.

#### 4.2.3. Hybrid Sampling

The datasets were balanced in the fourth and final experiment using a combination of undersampling and oversampling methods. As shown in Table 11, for the Morris power dataset, the first coupled algorithm was SMOTE and near miss. This method succeeded in balancing the Morris power dataset. The detailed values for the evaluation metrics are provided in Table 12. In the Morris power dataset, accuracy significantly reduced from 75% to only 59%.

On the other hand, the result of balancing the CICIDS2017 dataset with a hybrid technique is shown in Table 13. The performance of the binary classification model decreased. When ADASYN was combined with the near miss algorithm for hybrid balancing, a roughly similar result was obtained. The detailed values for the evaluation metrics are provided in Table 14. In the CICIDS2017 dataset, accuracy decreased from 93% to 93%.

**Figure 6:**
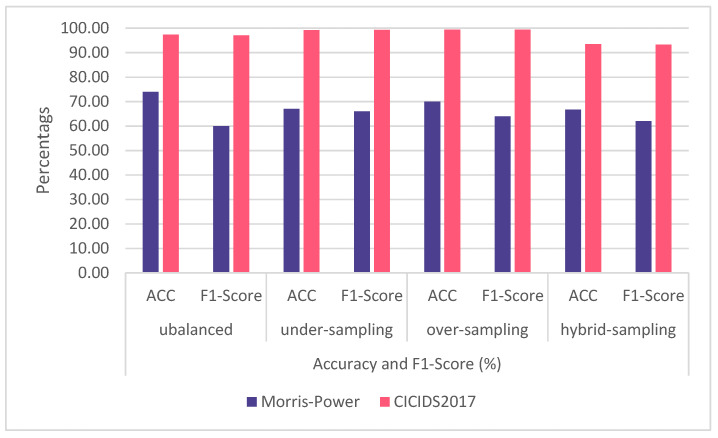
The F1-score and accuracy of both datasets in all four experiments.

The overall result is shown in Table 15 for the Morris dataset and Table 16 for the CICIDS2017 dataset. Figure 6 provides accuracy and F1-score, and it is clear from the pattern that when using the Morris power dataset, the CNN-LSTM model performs quite modestly. This is due to the dataset’s small size, which is only around 72,000. The outcome may differ if a machine learning algorithm is used. Deep learning techniques, however, need a bigger dataset. The results using the unbalanced CICIDS2017 dataset were satisfactory. When oversampling is used exclusively, the best outcome is obtained. Figure 6 demonstrates that the hybrid sampling method did not produce reliable outcomes. This is due to the dataset being distorted by the removal of records from the majority class and the addition of fake data to the minority class.

## 5. Conclusions

This research attempted to understand how dataset imbalances affected IDSs. To fully understand this effect, we conducted four experiments. balancing an unbalanced dataset by employing undersampling, oversampling, or a combination of both methods. Following the balancing step, the balanced dataset was used to train a binary classification model using CNN-LSTM. An imbalanced dataset does affect deep learning intrusion detection systems. This can be seen in Table 15 for the Morris power dataset and Table 16 for the CICIDS2017 dataset, the pattern seen is that undersampling and oversampling do improve the model’s performance. However, when the datasets were balanced with hybrid sampling, the evaluation metrics dropped significantly. This is due to the dataset being distorted by the process of adding and removing records. The dataset imbalance is not the only factor in the CNN-LSTM model performance, as the size of the dataset and the quality of the data are also significant factors. However, these factors are irrelevant to our research since the same dataset was used across experiments. We intend to create an intrusion detection system based on stable diffusion models in the future.

## Figures and Tables

**Table 1:** The binary classification with CNN-LSTM.

Dataset	No. of Records	Type of Records	No. of Classes
Morris Power	72,073	Normal and Attack	2
CICIDS2017	1,161,344	Normal and Attack	2

**Table 2:** Evaluation metrics for the unbalanced datasets.

Technique	ACC	Precision	Recall	F1-Score
Morris Power	73.63	73.22	74.84	58.06
CICIDS2017	98.42	98.03	98.12	98.44

**Table 3:** Balancing Morris power dataset with undersampling.

Technique	Before		After	
	Normal	Attack	Normal	Attack
Random	15,471	38,583	15,471	25,000
One Sided Selection	15,471	38,583	15,471	37,706
Near Miss	15,471	38,583	15,471	19,338

**Table 4:** Evaluation metrics for the Morris power dataset with undersampling.

Technique	ACC	Precision	Recall	F1-Score
Random	71.38	51	71	59
One Sided Selection	70.91	50	71	59
Near Miss	65.89	72.07	65.67	66

**Table 5:** Balancing the CICIDS2017 dataset with undersampling.

Technique	Before		After	
	Normal	Attack	Normal	Attack
Random Under Sampling	652,757	218,251	250,000	218,251
One Sided Selection	652,757	218,251	648,519	218,251
Near Miss	652,757	218,251	291,001	218,251

**Table 6:** Evaluation metrics for the CICIDS2017 dataset with undersampling.

Technique	ACC	Precision	Recall	F1-Score
Random	96.65	94	98	96
One Sided Selection	97.34	96.62	98.04	97.67
Near Miss	99.25	99.44	99.25	99.34

**Table 7:** Balancing Morris power dataset with oversampling.

Technique	Before		After	
	Normal	Attack	Normal	Attack
Random	15,471	38,583	30,866	38,583
SMOTE	15,471	38,583	34,724	38,583
ADASYN	15,471	38,583	32,425	38,583

**Table 8:** Evaluation metrics for the Morris power dataset with oversampling.

Technique	ACC	Precision	Recall	F1-Score
Random	71	51	71	59
SMOTE	70	65	70	64
ADASYN	71.37	64	71	61

**Table 9:** Balancing the CICIDS2017 dataset with oversampling.

Technique	Before		After	
	Normal	Attack	Normal	Attack
Random	652,757	218,251	652,757	476,512
SMOTE	652,757	218,251	652,757	522,205
ADASYN	652,757	218,251	652,757	457,547

**Table 10:** Evaluation metrics for the CICIDS2017 dataset with oversampling.

Technique	ACC	Precision	Recall	F1-Score
Random	99.63	99.04	99.78	99.41
SMOTE	99.47	99.43	99.49	99.46
ADASYN	93.62	92.37	99.18	93.25

**Table 11:** Balancing Morris power dataset with hybrid sampling.

Technique	Before		After	
	Normal	Attack	Normal	Attack
SMOTE and Near Miss	15,471	38,583	27,008	31,774
ADASYN and Near Miss	15,471	38,583	33,252	23,277

**Table 12:** Evaluation metrics for the Morris power dataset with hybrid sampling.

Technique	ACC	Precision	Recall	F1-Score
SMOTE and Near Miss	66.69	60	67	62
ADASYN and Near Miss	69.47	56	69	59

**Table 13:** Balancing the CICIDS2017 dataset with hybrid sampling.

Technique	Before		After	
	Normal	Attack	Normal	Attack
SMOTE and Near Miss	652,757	218,251	559,505	391,654
ADASYN and Near Miss	652,757	218,251	396,819	277,466

**Table 14:** Evaluation metrics for the CICIDS2017 dataset with hybrid sampling.

Technique	ACC	Precision	Recall	F1-Score
SMOTE and Near Miss	93.44	94.05	93	93.32
ADASYN and Near Miss	89.84	90.15	89.36	89.58

**Table 15:** Overall results for the Morris power dataset.

Technique	ACC	Precision	Recall	F1-Score
Unbalanced	73.63	73.22	74.84	58.06
Undersampling	65.89	72.07	65.67	66.09
Oversampling	70.23	65.54	70.32	64.18
Hybrid Sampling	66.69	50.31	67.52	62.49

**Table 16:** Overall results for the CICIDS2017 Dataset.

Technique	ACC	Precision	Recall	F1-Score
Unbalanced	98.42	98.03	98.12	98.44
Undersampling	99.25	99.44	99.25	99.34
Oversampling	99.47	99.43	99.49	99.46
Hybrid Sampling	93.44	94.05	93.00	93.32

## Data Availability

Not applicable.
